# Navigating the uncertainty: A novel taxonomy of vaccine hesitancy in the context of COVID-19

**DOI:** 10.1371/journal.pone.0295912

**Published:** 2023-12-21

**Authors:** Sekoul Krastev, Oren Krajden, Zoua M. Vang, Fernanda Pérez-Gay Juárez, Elizaveta Solomonova, Maya Goldenberg, Daniel Weinstock, Maxwell J. Smith, Lindsey Turk, Xingyan Lin, Ian Gold

**Affiliations:** 1 Integrated Program in Neuroscience, McGill University, Montreal, QC, Canada; 2 Faculty of Medicine, University of British Columbia, Vancouver, BC, Canada; 3 Civil Society and Community Studies, School of Human Ecology, University of Wisconsin-Madison, Madison, WI, United States of America; 4 Department of Philosophy, McGill University, Montreal, Quebec, Canada; 5 Neurophilosophy Lab, Division of Social and Transcultural Psychiatry, Department of Philosophy, McGill University, Montreal, Quebec, Canada; 6 Department of Philosophy, University of Guelph, Guelph, Ontario, Canada; 7 Faculty of Law, McGill University, Montreal, Quebec, Canada; 8 School of Health Studies, Faculty of Health Sciences, Western University, London, Ontario, Canada; 9 Dyson School of Applied Economics and Management, Cornell University, Ithaca, New York, United States of America; 10 Goldman School of Public Policy, University of California, Berkeley, Berkeley, California, States of America; University of Naples - Parthenope: Universita degli Studi di Napoli Parthenope, ITALY

## Abstract

Vaccine hesitancy remains a significant and evolving public health challenge. The COVID-19 pandemic has created a unique decision context with significant uncertainty caused by the novelty of the disease being targeted, unfamiliarity with the vaccines being offered, misinformation, and strong handed government measures. In an effort to extend our understanding of vaccine hesitancy to the high uncertainty decision environment presented by COVID-19, we present a novel taxonomy of the determinants of vaccine hesitancy, based on an inductive analysis of qualitative data gathered during the COVID-19 pandemic. We report on focus group data from a purposive sample of 18 Canadians with varying sociodemographic characteristics and COVID-19 vaccination attitudes. An inductive thematic analysis of this data reveals eight core themes related to vaccine hesitancy: values, trust, social environment, personal anecdotes, environmental fluctuation, prior knowledge, perceived risk & systems of care. We explore these core themes as well as 25 sub-themes, contrasting them with previous models of vaccine hesitancy and suggesting potential strategies for public health professionals.

## Introduction

It is estimated that 14.4 million COVID-19-related deaths were prevented globally by vaccines between December 2020 and December 2021 alone [1]. However, despite being one of the most powerful and safe public health tools we have access to, vaccines are still not universally accepted. In fact, a study of over 140,000 people in more than 140 countries found that 79% of people believe vaccines are safe and 84% think they are effective [2]. These attitudes are reflected in real world behaviors—namely, vaccine hesitancy, i.e. “a delay in acceptance or refusal of vaccination despite availability of vaccination services” [3]. Despite a wealth of evidence showing the safety and efficacy of vaccines, only 75% of the global population [4] are willing to accept being vaccinated. In the context of COVID-19 vaccines, acceptance rates vary significantly around the world—for example, 97% in Ecuador, 54.9% in Russia and 23.6% in Kuwait [5]. In Canada, vaccine acceptance rates in the midst of the pandemic were at 75% [6]. While we can say that vaccines have high efficacy, as measured by their success in those who take them, the varied uptake has meant that their effectiveness, as measured by actual real world outcomes and therefore composed of efficacy in combination with uptake, is limited [7].

### Why are people vaccine hesitant?

Vaccine hesitancy is heterogenous and context-dependent in a variety of ways. Firstly, it is important to note that while the current literature on vaccine hesitancy is focused on individuals making decisions about their own vaccination behaviors, pre-COVID vaccine hesitancy research largely focused on pediatric decisions, i.e. parents making decisions about their children [8]. While these two constructs clearly differ, research has shown that there is a significant relationship between vaccine hesitancy in the two contexts. For example, Soares and colleagues showed an association between refusal to take the flu vaccine and intention to refuse the COVID-19 vaccine [9, 10]. Secondly, it is worth noting that the exact beliefs and attitudes that underlie vaccine hesitancy shift between different diseases and even between different vaccines targeting the same disease. For example, Brewer and colleagues [11] found that parents have different levels of hesitancy and reasons for being hesitant depending on the type of vaccine concerned (in their work, MMR versus HPV). Furthermore, work by Merkley & Loewen [12] involving 6200 participants showed even within vaccines for a single disease (COVID-19), the same vaccine elicited differential levels and sources of hesitancy. Thirdly, even in cases where research is focused on a single vaccine targeting a single disease, vaccine hesitancy may not mean the same thing from one participant to another. For example, Noni MacDonald [3] has proposed that vaccine hesitancy can better be understood along a continuum that contains various attitudes such as “refuse all with conviction”, “refuse all, but unsure”, “accept some, refuse some, delay vaccination”, “accept with doubts & concerns” and “accept all with confidence”. Finally, even when people are vaccine acceptant, it may be for different reasons, unrelated to the vaccine itself (e.g., confidence in vaccine efficacy). Alfano and colleagues [13] showed that positive attitudes toward vaccines can be for individualistic or altruistic reasons.

Given how complex vaccine hesitancy is, a large amount of past research has focused on understanding its antecedents, including individual differences in vaccine hesitancy such as demographic correlates. For example, in the context of COVID-19, being a woman, being 50 years or younger, being single, being unemployed, and living in a household with five or more people, were factors associated with increased vaccine hesitancy [14]. While findings like these allow us to understand which groups might present the biggest challenge for vaccination in a public health context, they do not necessarily provide an effective strategy for targeting those groups because they shed little light on the motivating factors underlying vaccine attitude formation. For this reason (among others), recent research has started to tackle the psychological correlates of vaccine hesitancy [11, 15, 16]. For example, Betsch and colleagues [15] showed, from a group of 1000 German participants, that confidence, complacency, constraints, calculation, and collective responsibility are key psychological antecedents of vaccination. Hornsey and colleagues [16] used a sample of over 5000 people to show strong associations between vaccine hesitancy and conspiratorial thinking, reactance, disgust towards blood and needles, and individualistic world views.

Although empirical research is invaluable in allowing us to gain a better understanding of factors related to vaccine hesitancy, previous literature on behavior change [17, 18] has shown that understanding a decision environment in a holistic and mechanistic manner is beneficial to engendering behavior change. In recognition of this, a number of models have been proposed to explain causal factors related to vaccine hesitancy—some specific to vaccine hesitancy and some broadly related to uptake of health behaviors, but all applicable to vaccine uptake. These models, which we review below, have an advantage over questionnaire-style empirical reports of factors related to vaccine hesitancy in that they base themselves on large bodies of empirical findings and thus purport to map the terrain in order to create more evidence-based directions for future hypotheses and interventions. In an effort to better understand this terrain, we review existing models related to vaccine hesitancy and then use these models to create a search space for a thematic analysis of qualitative data from pandemic-era focus groups.

### Frameworks focused on health behaviors

#### Health Belief Model

The Health Belief Model (HBM) was developed in the 1950s by the US Public Health Service and remains one of the most widely used frameworks for assessing and predicting the uptake of health related behaviors [19]. The HBM suggests that behaviors such as getting a vaccine are linked to a person’s perception of factors such as the severity of the disease, their susceptibility to it, the benefits of taking preventive action, and the barriers to taking that action. Changes have been made to the HBM over the years to reflect advances in behavioral science, such as the inclusion of self efficacy [20].

#### Theory of Planned Behavior

The Theory of Planned Behavior (TPB) was developed in the 1990s by Icek Ajzen [21] and suggests that intentions to perform a behavior, and consequently, the uptake of a behavior, can be accurately predicted from a person’s attitudes toward the behavior, subjective norms around the behavior and perceived behavioral control over the behavior. The TPB differs from the HBM in that it adds a significant social layer to the causal factors behind a behavior and has therefore been an instrumental addition in healthcare contexts, explaining myriad behaviors [22]. In the context of vaccine uptake in particular, evidence suggests that while there is significant overlap between the TPB and HBM, the TPB tends to consistently outperform when used to predict real-world behaviors [23].

### Taxonomies of uncertainty

Another set of frameworks used to explain health related behaviors comes from the study of uncertainty. The link between uncertainty (i.e., the subjective feeling of ignorance about something) and vaccine hesitancy has been well documented. In fact, past studies have shown that uncertainty is one of the most critical factors associated with vaccine hesitancy [24, 25]. In a study that looked at COVID-19 vaccination efforts, Courbage and Peter [26] found that reducing uncertainty about the vaccine reliably promotes vaccination.

Vaccine decisions must often take place in an inherently uncertain environment with shifting and often conflicting recommendations. This has been all the more true during the COVID-19 pandemic, where the time horizon of recommendation changes was sometimes weeks or days [27]. Past research suggests that this type of scientific uncertainty creates distrust in scientists and public health recommendations [28], which in turn has been shown to result in low confidence in vaccines [29] and lower vaccine uptake [30]. Indeed, perceptions of uncertainty affect trust toward all disease-related information [31], particularly in individuals with lower tolerance for ambiguity and risk. Uncertainty has also been shown to make people more pessimistic about disease treatment [32] and perhaps most importantly, lower intentions to engage in health-promoting behaviors [33]. A number of efforts have been made to taxonomize the types of uncertainty relevant to healthcare decisions in an effort to better understand and support patient experience with the overall goal of improving decision making and clinical outcomes.

#### Varieties of uncertainty in healthcare

Han and colleagues [34] proposed a taxonomy (Table 1) that separates uncertainty according to source and issue. Within sources of uncertainty lie ambiguity, complexity, and probability. Furthermore, within issues of uncertainty lie scientific, practical, and personal issues, each of which have been broken down into two or more categories.

**Table 1:** Han et al.’s (2011) suggested taxonomy of uncertainty.

Sources of Uncertainty	Issues of Uncertainty
Ambiguity	Scientific
Complexity	Practical
Probability	Personal

#### Model of Uncertainty within Complex Healthcare Settings

In response to the above proposed taxonomy, Pomare and colleagues [35] posited that uncertainties do not occur in isolation, as they argued the above taxonomy implies. The research team examined the empirical healthcare literature to see whether or not Han and colleagues’ taxonomy is applicable to the wide range of sources of uncertainty experienced by healthcare providers in the healthcare space. After examining the 94 articles that passed their criteria, their research revealed that Han and colleagues’ taxonomy [34] would have benefitted from two new sources of uncertainty: systems uncertainty and ethical uncertainty. Furthermore, Pomare and colleagues’ new model highlights the overlapping nature of uncertainty which they argue is a necessity and more realistic than disparate and unrelated sources of uncertainty. The taxonomy they suggest (Table 2), Model of Uncertainty within Complex Healthcare Settings (MUCH-S) splits healthcare uncertainty into three main (and overlapping) categories:

**Table 2:** Pomare et al.’s (2018) suggested taxonomy of uncertainty.

Personal Uncertainty	Scientific Uncertainty	Practical Uncertainty
Psycho-social	Diagnosis	Structure of care
Existential	Prognosis	Processes of care
Ethical	Causal explanations	Systems
	Treatment options	

#### Vaccine-specific frameworks

In an effort to refine the focus of causal factors to specifically target vaccine uptake, a number of frameworks have been proposed that are vaccine specific.

#### 3C model

The SAGE working group was established by the World Health Organization in 2012 to propose a behavioral model that categorizes factors that influence vaccine uptake [3]. The 3C Model was one of the outputs of this working group. This model suggests that vaccination decisions depend on three core factors: *complacency*, which exists when someone does not feel that the risks of the disease warrant taking action; *convenience*, which relates to the availability, accessibility and quality of service related to vaccination; *confidence*, which refers to the safety and effectiveness of the vaccines, the reliability and competence of health services, and the motives of the institutions behind them.

#### 5A model

A broader taxonomy for determinants of vaccine uptake was proposed in 2016 by Thomson and colleagues [36], based on a review of 43 studies related to vaccine hesitancy. According to this model, vaccine uptake is influenced by: *access*, the extent to which a person can reach the vaccine; *affordability*, the ability of an individual to afford (in both financial and non-financial terms) the vaccine; *awareness*, the extent to which an individual understands the need for the vaccine; *acceptance*, the extent to which individuals accept the vaccine; and *activation*, the extent to which individuals receive a contextual cue urging them to get vaccinated [36].

#### 5C Model

Betsch and colleagues [15] revisited the 3C model using an empirical approach focused on a German population. Their intention was to create a novel taxonomy that focuses on more psychological antecedents of vaccine uptake. Their resulting 5C taxonomy replaced complacency from the 3C model with constraints and suggested the addition of two factors: *calculation*, which refers to engagement in extensive information searching about vaccines; and *collective responsibility*, which refers to willingness to protect others by getting vaccinated.

The dominant models reviewed above add a more holistic and mechanistic explanatory level to the vaccine hesitancy landscape. They supplement empirical research by proposing mutually exclusive and collectively exhaustive categories of factors in a way that can better drive future hypothesis-generation and intervention design. As such, these models have been instrumental in providing insight into why people might not be engaging in vaccination. However, despite their effectiveness in a general vaccine hesitancy context, they have all been developed in contexts that have less inherent uncertainty than the COVID-19 pandemic. Notably, the pandemic introduced a combination of factors that made vaccination decisions more difficult: the novelty of the disease being targeted, the burden of government interventions being imposed, a significant amount of politicization and misinformation about vaccines, unfamiliarity with the vaccines being offered, and the rapid rate of spread and evolution of the disease. We use the phrase “high uncertainty vaccination decisions” to refer to decisions where these factors are present.

While efforts have been made to verify the extent to which the HBM, TPB, 3C, 5A and 5C models apply in high uncertainty vaccination decisions–resulting in a suggestion that the HBM and 3C are most applicable–the conclusion has been that a new model which possibly combines features of past models might be most apt [37]. Indeed, as we have seen above, past research suggests that vaccine hesitancy can differ significantly between diseases [11] and even more so between COVID-19 and non COVID-19 contexts [10].

These important differences suggest that a model of vaccine hesitancy that specifically relates to high uncertainty vaccination decisions may be an important public health tool. In order to construct this model, we draw on empirical evidence gathered during the COVID-19 pandemic. In particular, we had the following research objectives:

1. To identify the multitude of determining factors related to COVID-19 vaccine acceptance based on structured qualitative analysis of focus group data generated during the COVID-19 pandemic.
2. To develop a theoretical model of the determinants of vaccine hesitancy in the context of high uncertainty vaccination decisions based on the factors identified.

## Methods

In order to meet these objectives, we collected data from focus groups with 18 adults, used an inductive thematic analysis to identify factors related to vaccine hesitancy, and then used insights from the models reviewed above to further refine and contextualize the emerging themes. This approach allowed us to propose a novel model of vaccine hesitancy adapted to a COVID-19 context. All methods and protocols were carried out in accordance with relevant guidelines and regulations and were approved by McGill University’s Institutional Review Board (Ethics Approval ID: 22-10-064). In addition, informed consent was obtained from all subjects prior to data collection.

### Data collection methods

Research was carried out in collaboration with Environics Research (https://environics.ca/), a Canadian polling and research firm, for the purpose of data collection. Participants were recruited using a purposive sampling strategy between June 23rd, 2021 and August 4th, 2021 based on their self-identified vaccination status and attitudes in a national survey of COVID-19 experiences. Survey data, which we do not report on in this study, was collected from 1541 Canadians in April and May, 2021, during which time participants were asked if they were interested in participating in follow-up focus groups. Among the 758 individuals who agreed (49%), we selected 538 profiles specifically fitting a sampling strategy selected to include a balanced urban/rural split, a wide range of ages, ethnicities, geographical locations and income ranges. We contacted them by e-mail and 18 individuals agreed to participate in the focus groups. Data was collected in focus groups that were carried out virtually between September 7th and September 9th, 2021. Given that COVID-19 vaccines became available in Canada in December 2020, and were widely available by Summer 2021, focus groups were carried out at a time where vaccination decisions were top-of-mind for participants. A total of four focus group sessions were conducted, each lasting between 60 and 67 minutes with a mean length of 64.

The four focus groups consisted of participants with different vaccination statuses. Group 1 participants were unvaccinated individuals who indicated that they would either not get vaccinated at all or would wait a while before deciding. Group 2 consisted of vaccinated individuals who were initially hesitant about receiving a COVID-19 vaccine but ultimately chose to get vaccinated. Groups 3 and 4 consisted of young adults with mixed vaccination statuses.

The four groups included 18 adults, consisting of 13 women and 5 men. Table 3 illustrates the primary sociodemographic characteristics of the participants, including their vaccination status at the time of the focus groups. The age of the participants ranged from 26 to 68, with an average age of 44. All participants were born in Canada and identified themselves as either white (67%) or Indigenous (33%). They were from six provinces across eastern, central, and western Canada, with the majority residing in Ontario. The participants were geographically dispersed, with some living in rural areas and some in urban areas. Ten of the female participants were mothers, while none of the male participants had children. Half of the participants were low-income, and most had at least a high school degree. To maintain confidentiality, pseudonyms were assigned to the participants. Focus groups were moderated by two researchers at Environics Research: a male researcher, who has nearly 15 years experience in the healthcare industry and market research and a female researcher with over six years experience conducting qualitative research, including in virtual platforms. Two researchers from the university investigator team were also present during focus group sessions but did not moderate. Participants were asked to sign a consent form which clearly indicated the purpose of the study and were told the identities of the interviewers. The focus groups were conducted using the guide available in the S3 Appendix, which was designed by the authors, and a recording was made, which was later transcribed. No repeat interviews were carried out and transcripts were not returned to participants for correction.

**Table 3:** Sociodemographic characteristics and vaccination status of focus group participants.

	Age	Sex	Race/Ethnicity	Province	Education	Low Income Status	Received COVID-19 Vaccine?
**Group 1**							
Brooke	36	F	Indigenous	BC	Some college	Yes	No
Eleanor	43	F	White	NB	HS degree	Yes	No
Heather	68	F	White	ON	Less than HS	No	No
William	44	M	White	MB	HS degree	Yes	No
Melanie	52	F	Indigenous	AB	Some college	Yes	No
Billy	64	M	Indigenous	ON	Some college	Yes	Yes
**Group 2**							
Julie	58	F	White	SK	HS degree	No	Yes
Patrick	53	M	White	ON	Some college	Yes	Yes
Brianna	63	F	White	ON	HS degree	Yes	Yes
Joy	49	F	Indigenous	ON	HS degree	No	Yes
Esther	60	F	White	ON	Less than HS	No	Yes
Clara	26	F	White	ON	Some college	Yes	Yes
**Groups 3 & 4**							
Lorry	30	F	White	ON	Bachelor’s degree	No	Yes
Ethan	26	M	White	QC	Some college	No	No
Molly	26	F	White	ON	Bachelor’s degree	No	No
Janet	26	F	Indigenous	ON	HS degree	Yes	No
Charles	31	M	White	AB	Bachelor’s degree	Yes	No
Elise	28	F	Indigenous	AB	Bachelor’s degree	Yes	Yes

### Data coding & analysis

Focus group recordings were transcribed verbatim. MAXQDA Analytics, a qualitative analysis software, was used to aid in the analysis of focus group transcripts. The software was in a manual capacity—i.e. no automated features were used to generate codes or thematic categories. Using MAXQDA enabled us to manually organize the transcript into the codes using the process detailed below and provided visualizations of critical elements using various built-in tools.

The study utilized an inductive approach to conduct a thematic analysis (Braun & Clarke, 2006), a process where researchers actively engage in the knowledge production process and consider their subjectivity as a valuable resource during the coding and theme development phases. This approach allowed for a systematic way of processing qualitative information using coded text and enabled us to provide a detailed and nuanced analysis of participants’ attitudes and behaviors toward COVID-19 vaccines. The steps we took, proposed by Braun and Clarke [38], are data familiarization, code formulation, generation of themes, themes review, defining and naming themes, and report formation. A single coder, XL, was used throughout the process to ensure consistency in the coding, with other members of the research team providing guidance on the coding tree. The coder has a professional and academic background in public health and qualitative research, specifically focused on vaccine hesitancy. Participants were not asked to provide feedback on the findings.

1. **Data cleaning & familiarization:** We transcribed the data based on audio recordings, and imported the transcripts into MAXQDA, then completed the necessary pre-processing steps: removing identifying information & formatting the data for analysis. We read and re-read the transcripts end-to-end in order to familiarize ourselves with the contents and generate initial ideas for a search space.
2. **Code formulation:** Using the generated ideas as well as past models used in vaccine hesitancy as a starting point for the search space, a coder read through all of the transcripts, coding representing sections related to vaccine hesitancy in a systematic fashion across the entire dataset–both sections related to the search space and novel themes were identified, with novel themes focused on expressions of hesitancy around vaccination decisions. Memos were written down to keep track of the condensed information. A codebook was developed based on this process, and then applied to the data using MAXQDA’s coding function. The data were then segmented into coded units based on the codes applied.
3. **Generation of themes:** The coder reviewed the coded data and refined the codes as necessary. New codes were added, and existing codes were merged or split based on the patterns that emerged from the data. The cycle was repeated several times to narrow down the number of codes and categorize them into identifiable themes. The codes were then analyzed and grouped into three central themes as stated in the results section.
4. **Themes review:** The complete interview data were re-read to validate that the themes were gathered in an accurate and representative way. MAXQDA was used to identify patterns within the data, and these were used to draw conclusions.
5. **Defining and naming themes:** The reviewed themes were then conceptualized and assigned clear definitions in the S1 Appendix, and memos were finalized within MAXQDA to ensure data transparency and reproducibility.
6. **Report formation:** several vital statements and features representing the data were extracted to showcase the resulting outcomes both as statements in the form of ideas and feelings and visual representations using interconnections between codes as seen in the S2 Appendix.

In order to ensure rigor in our qualitative research process and validation of our findings, we used an iterative thematic analysis process based on established analytical tools [38]. Furthermore, we recruited a diverse sample of individuals and maintained records of our processes, creating a MAXQDA codebook. Finally, we engaged in self-reflection throughout the process to better understand personal biases and report on potential sources of these biases in the limitations section.

## Results

Our thematic analysis revealed 8 core themes and 25 sub-themes, all of which could be attributed to one of the three broad categories: scientific, personal, and practical. Our coder determined that these broad categories, which also appear in Pomare and colleagues’ [35] taxonomy of uncertainty, best captured the broad categories of vaccine hesitancy in the data. Table 4 below provides more information about the themes that were generated during the coding process.

**Table 4:** Vaccine hesitancy themes identified in the data.

Category	Themes	Sub-Themes	Frequency	% of individuals
**Personal**	**Values**	Political Compass	5	22.2%
		Autonomy	29	33.3%
	**Trust**	Trust in the medical profession	26	38.9%
		Trust in the medical caretaker	9	44.4%
		Trust in Government	43	72.2%
		Trust in Pharmaceutical Companies	4	5.6%
		Trust in Media	18	55.6%
	**Social Environment**	Pressure from Close Community	18	55.6%
		Pressure for Broad Community	3	16.7%
		Pressure from Society	15	44.4%
	**Personal Anecdotes**	Personal Experience	3	11.1%
		Experience of those close by with vaccines	12	27.8%
		Hearsay	16	44.4%
**Scientific**	**Perceived** **Risk**	Short-term side effects	31	88.9%
		Long-term side effects	11	44.4%
		Risk of serious symptoms or death due to COVID-19	13	38.9%
	**Prior** **Knowledge**	Effectiveness of Vaccine	18	72.2%
		Understanding of Vaccine Function	4	22.2%
		Belief that natural immunity is better	3	11.1%
		Information Overload	15	44.4%
	**Environmental Fluctuation**	Possible evolution of the Vaccine	13	44.4%
		Flawed measurement of the Vaccine	22	50.0%
		Possible evolution of the Disease	4	22.2%
		Flawed measurement of the Disease	4	16.7%
**Practical**	**Systems of Care**	Being out of commission	7	27%

The S2 Appendix provides a full list of these themes, along with the supporting data extracts from the focus group transcripts. In the following sections, we provide more detailed discussion of these themes, focusing on illustrative examples that support each sub-category.

### Personal

#### Value

In our study, values were a major source of vaccine hesitancy, with participants expressing strong feelings around the right to choose and a clash between their personal value system and public health agencies’ approach to vaccination. Nine participants voiced similar concerns. For example, one participant noted that people "should be taken on [their] free will [to make vaccination decisions]," and that "it’s everyone’s choice what they feel is best for them." Another participant, who had already been vaccinated, stated that "it’s everybody’s decision, whether they want to get [vaccinated] or not…[I wish] everybody would. It might bring cases down more, less death, less sick people. But it’s up to everybody." Another participant called for accepting people for who they are and their decision to vaccinate or not. "It’s your decision…we should accept people for who they are [and] embrace that." As a third participant put it:

But… how they were restricting… access to certain things, and… if we don’t have the vaccine… which I really didn’t like… for me that… makes me… less likely to get the vaccine, because… it’s not really acceptable to me.*—Ethan*, *26-year-old*, *unvaccinated*

Words like “condescending”, “passive-aggressive”, and “pushy and exclusive” were frequently used by participants when discussing messages about COVID-19 vaccines, which evoked feelings of “othering” and stigmatization. A participant felt as if she was being alienated from society for not getting vaccinated. "When you press people, it’s going to make them push back 10 times as hard," she stated.

#### Trust

Throughout the focus group discussions, participants expressed varied views on the trustworthiness of medical professionals, health providers, the pharmaceutical industry, and government officials in the development, approval, and distribution of COVID-19 vaccines. Participants, who were vaccinated or intended to get vaccines in the future, cited medical professionals like Dr. Anthony Fauci as trustworthy sources of COVID-related information, who have a significant effect on individuals’ vaccination decisions. For example, one participant, who was not vaccinated yet but planned to get one eventually, took Dr. Fauci’s suggestions as “the gold standard” during his decision-making process.

However, not everyone shared the same confidence in medical professionals. Patrick found conflicting views from medical professionals, prompting him to turn to what he considered to be independent news companies that compared different sources of information to ensure the reliability of the COVID-related information he received. Similarly, one participant felt that legitimate sources of information were being censored to hide true risks behind the vaccine:

So those kinds of things just are raising major red flags for me, and I feel like from the doctors that have spoken out, Dr. Byron Biddle, Dr. Christina Parks, Dr. Sunetra Gupta… they’re… sharing valid things and they’re being censored [by the government], and that should raise red flags for everybody.*—Janet*, *26-year-old*, *unvaccinated*

#### Social environment

Social factors emerged among participants, in most cases linked to the influence of others, including friends, family, or community members. It manifested in various forms, such as social pressure to avoid vaccination or the impact of misinformation and conspiracy theories shared by others.

As noted by past research, strong ties represented a significant source of vaccine hesitancy. In some cases, this was in the direction of non-vaccination. In other cases, it was in the direction of vaccination. For example, one participant noted:

she refused to get vaccinated, but I said to her, look, I said, your father and I both work where we are in contact with people 24/7, because I work at a fast food restaurant, my husband works at a lumberyard, he’s a truck driver. And I said, we don’t know if we’re going to end up coming in contact with someone that’s not vaccinated, that has it. So she finally got vaccinated.—*Esther*, *60-year-old*, *vaccinated*

While personal-level social pressure was generally effective, institutional pressure often backfired. For example, COVID-19 vaccine campaigns that contained messaging such as “don’t be selfish, vaccinate to save others” were not well received by unvaccinated participants. Unvaccinated participants explained that the messaging made them feel shamed and separated them socially and morally from the vaccinated population. As one 26-year-old unvaccinated participant stated, the messaging implies that “you’re not a good person…you’re not protecting your family, maybe you even want them dead [if you choose not to get vaccinated]…it’s not ‘we’re in this together’ messaging.” Another unvaccinated participant, shared that “I find the ads are not only pushing it but they’re making people who don’t choose it feel like they’re outcasts and there are people who have had a lot of bad backwash from that I’ve heard of people actually being bullied because they don’t get it.”

#### Personal anecdotes

Our analysis showed that participants’ hesitancy around COVID-19 vaccines was related to anecdotes derived from their personal experience as well as the experience of their strong ties. For example, as two participants noted:

My sister had terrible side effects, vomiting for two days just you know where she needed somebody to come and take care of her and that was really worrisome.*—Molly*, *26-year-old*, *unvaccinated*

I actually had a neighbor who had the Pfizer vaccine, and he had really bad side effects for a day or two after, and you know it made me kind of pause.*—Brooke*, *36-year-old*, *unvaccinated*

Another participant was also hesitant about vaccination after her friend contracted COVID-19 despite being vaccinated.

I was… iffy about it, because my one friend, he got… the needle. And then he still got COVID. So like, I didn’t know, maybe he got COVID from… the shot.*—Clara*, *26-year-old*, *vaccinated*

### Scientific

#### Environmental fluctuation

In our focus group discussions, participants expressed a pervasive sense of uncertainty about the progression of COVID-19 and COVID-19 vaccines. Many of their concerns were driven by the unpredictable nature of the disease and its potential evolution, which left some feeling uneasy, even if they had been vaccinated. Two participants, for instance, shared concerns about new variants and the potential for more contagious strains to emerge:

Now… we have this fourth variant, or this one new variant… it’s mutating, and that’s…really scary… and they’re saying that this new variant is more contagious but less harmful… that doesn’t give me comfort.*—Patrick*, *53-year-old*, *vaccinated*

I’m worried about how big COVID’s going to get with the new variants; I’m worried about our society as a whole right now.*—Elise*, *28-year-old*, *vaccinated*

#### Prior knowledge

The changing nature of the disease and its vaccines led to ambiguity and confusion in COVID-related knowledge. One common confusion was around the effectiveness of vaccines. Two participants expressed concerns regarding the vaccines’ ability to prevent infection and the potential for increased side effects with each booster shot. One shared her confusion around the effectiveness of vaccines:

I agree at least a couple years, because then at least they’d have had a chance to test it on a variety of different people with different conditions.—*Melanie*, *52-year-old*, *unvaccinated*

#### Perceived risk

One of the most commonly discussed sources of vaccine hesitancy in our data was related to participants’ risk perceptions around vaccines. Second-hand stories of individuals experiencing severe side effects or even death after vaccination were prevalent. Two participants both heard that “people were dying or people were having really bad side effects”.

Yes, I heard that people were dying getting the shot, after they got the shot that they were dying. So I was quite scared to get it.*—Joy*, *49-year-old*, *vaccinated*

“If one is saying that there’s a chance of getting a blood clot. Well, how can they say that the other two are going to be okay” one participant concluded. Their shared fear was reinforced by news that “AstraZeneca [has] already been taken off the market because of very serious side effects.”

Another participant also expressed concerns that vaccines could be harmful to people with respiratory issues.

I’ve heard that it can be really bad for people that have respiratory issues… I have asthma, I have very bad allergies, I’ve had breathing problems.—*Eleanor*, *43-year-old*, *unvaccinated*

### Practical

#### System of care

Participants expressed concerns around the effects of being unvaccinated on access to public care systems. For example, one person shared a personal experience of being unable to access home care after having a toe amputated because he was not vaccinated.

Back on Labor Day of last year, I actually ended up in the hospital for unrelated—I had to have a toe amputated due to it being infected… after I got out of the hospital, I had a home care worker coming into my home and they basically said, because you’re receiving home care, they’re strong—they really wanted me [to get vaccinated]… otherwise I would lose my home care worker once every few days to help me with some things and change the bandages. So I knew that I had to have it because of, you know, the other problem I was dealing with.—*Patrick*, *53-year-old*, *vaccinated*

Other participants shared concerns about the broader costs of remaining unvaccinated on aspects of their lives such as access to employment, accessibility of public services and travel. For example, Lorry and Janet expressed concerns about people getting fired because of their medical decisions. In addition, Lorry mentioned “the obstacles of not being able to travel… not going to concerts and the giveaways seem small to [me]”, and “the only big obstacle that [I see] would be [my] job.”

## Discussion

### Proposed taxonomy

Our analysis of past frameworks suggests that, while they are useful in a non-pandemic context, a number of important gaps exist that limit their usefulness in a COVID-19 context and potentially in the context of future vaccine-preventable pandemics where high uncertainty vaccination decisions occur. Given the demonstrated effectiveness of targeted messaging in increasing vaccine uptake, overcoming these limitations is particularly important in order to inform the design of effective public health messaging. In an effort to address the gaps in existing models, we propose a novel taxonomy of vaccine hesitancy that is grounded in our focus group findings, is specific to COVID-19, and is potentially applicable to future vaccine-preventable pandemic contexts. This taxonomy is based on the themes that emerged from our analysis of the focus group data. This means that it differs in approach from past taxonomies, a large part of which were constructed from literature reviews of determinants of vaccine hesitancy. Given that the studies included in these literature reviews did not necessarily aim to uncover the entire landscape of vaccine hesitancy determinants but rather focused on specific hypotheses, our taxonomy represents a more naturalistic approach that is unconstrained by prior hypotheses. Our taxonomy—which we refer to as the High Uncertainty Vaccination Decision (HUVD) taxonomy—is presented in Table 5.

**Table 5:** The High Uncertainty Vaccination Decision (HUVD) taxonomy (full definitions of themes and sub-themes provided in the S2 Appendix).

Personal	Scientific	Practical
Values	Environment Fluctuation	Systems of care
Trust	Prior Knowledge	
Social Environment	Perceived Risk	
Personal Anecdotes		

Notably, we found that focus group participants had strong feelings about their right to choose whether or not to be vaccinated. With these feelings, came a resistance to what is perceived as external pressure and restrictions tied to vaccination. However, while institutional pressures were expected to backfire, likely driven by low levels of trust, the influence of social circles was more effective in promoting vaccination. With regard to the development of the vaccines, even non-hesitant individuals felt a large degree of uncertainty and confusion about effectiveness and potential side effects, suggesting that the unique timeline in which the COVID-19 vaccines were developed may have contributed to perceived risk.

### Comparison to past taxonomies

Our proposed HUVD taxonomy focuses on determinants of vaccine hesitancy in a COVID-19 context. Therefore, it differs in a number of ways from other models we have summarized in Table 6.

**Table 6:** The High Uncertainty Vaccination Decision (HUVD) taxonomy compared to previous models used in vaccine hesitancy.

HBM	TPB	Han et al.	Pomare et al.	3C Model	5A Model	5C Model	HUVD Taxonomy
*Perceived Susceptibility*	*Attitudes*	*Ambiguity*	*Psycho-social*	*Complacency*	*Access*	*Convenience*	**Values**
*Perceived Severity*	*Subjective Norms*	*Complexity*	*Existential*	*Convenience*	*Affordability*	*Confidence*	**Trust**
*Health Motivation*	*Perceived Control*	*Probability*	*Ethical*	*Confidence*	*Awareness*	*Constraints*	**Social Environment**
*Perceived Benefits*		*Scientific*	*Diagnosis*		*Acceptance*	*Calculation*	**Personal Anecdotes**
*Perceived Barriers*		*Practical*	*Prognosis*		*Activation*	*Collective Responsibility*	**Environment Fluctuation**
		*Personal*	*Causal Explanations*				**Prior Knowledge**
			*Treatment Options*				**Perceived Risk**
			*Structure of care*				**Systems of Care**
			*Processes of care*				
			*Systems*				

In an effort to better understand the differences and similarities between our proposed taxonomy and previous taxonomies, we created the following comparison, which aims to illustrate which categories in our taxonomy might be most relevant for each category of previous taxonomies. One notable difference between our proposed taxonomy and existing taxonomies is that previous taxonomies have a much greater focus on various aspects of what we call “systems of care”—i.e. on the actual mechanics of delivering the vaccine. While systems of care are also critical in the context of COVID-19, participants in our focus groups were far more likely to bring up factors relating to uncertainty, trust and institutional pressures. These factors, which reflect the high uncertainty environment of the COVID-19 pandemic and vaccine development lifecycle, are likely to also be more relevant in future contexts where vaccine-preventable diseases emerge and require fast action. In Table 7, we further illustrate the differences between our proposed taxonomy and past taxonomies.

**Table 7:** A mapping of categories in the HUVD taxonomy to those of previous models.

HBM	Relevant Categories from our Taxonomy
Perceived Susceptibility	Perceived Risk, Prior Knowledge
Perceived Severity	Perceived Risk, Prior Knowledge
Health Motivation	Values, Prior Knowledge
Perceived Benefits	Prior Knowledge, Trust
Perceived Barriers	Systems of Care
**TPB**	
Attitudes	Values, Trust
Subjective Norms	Social Environment, Trust
Perceived Control	Systems of Care, Trust
**Han et al.**	
Ambiguity	Environment Fluctuation, Perceived Risk
Complexity	Environment Fluctuation, Prior Knowledge
Probability	Perceived Risk
Scientific	Prior Knowledge, Trust
Practical	Systems of Care
Personal	Personal Anecdotes, Trust
**Pomare et al.**	
Psycho-social	Social Environment, Trust, Values
Existential	Values
Ethical	Values, Trust
Diagnosis	NA (Not Relevant to Vaccination)
Prognosis	NA (Not Relevant to Vaccination)
Causal Explanations	NA (Not Relevant to Vaccination)
Treatment Options	NA (Not Relevant to Vaccination)
Structure of care	Systems of Care
Processes of care	Systems of Care
Systems	Systems of Care
**3C Model**	
Complacency	Perceived Risk, Values
Convenience	Systems of Care
Confidence	Trust, Social Environment, Prior Knowledge
**5A Model**	
Access	Systems of Care
Affordability	Systems of Care
Awareness	Prior Knowledge, Social Environment
Acceptance	Trust, Prior Knowledge, Social Environment
Activation	Trust, Systems of Care
**5C Model**	
Convenience	Systems of Care
Confidence	Trust, Social Environment, Prior Knowledge
Constraints	Systems of Care
Calculation	Prior Knowledge, Trust
Collective Responsibility	Values

Importantly, previous models focus on particular aspects of what our data showed to be relevant in a COVID-19 vaccine hesitancy context, but none of them cover all of the categories we identified. For example, the HBM focuses on individual perceptions and motivations but does not tie them to the social environment. The TPB connects better to social environments but does not capture (or too broadly captures) the scientific and environmental uncertainty that are inherent in high uncertainty vaccination decisions. The uncertainty taxonomies from Han and colleagues and Pomare and colleagues capture a wider breadth of categories but in a way that is too far removed from a vaccine context. Finally, the 3C, 5A and 5C models are more narrowly focused but tend to be more granular around systems of care (which our data suggests is not a significant driver) and less granular around confidence and acceptance, which limits the usefulness of these categories.

Overall the differences between our HUVD taxonomy and past taxonomies are reflections of the unique decision environment of the COVID-19 pandemic. A number of factors have come together to create this environment, such as: the unprecedented speed of vaccine development; novel vaccine technologies; global scale of the illness; large amounts of media attention; large amounts of misinformation and disinformation; political polarization; unpredictable evolution of the disease and variants of concern; lockdowns and restrictions; debate about natural immunity induced by infection. Given factors like these, it is unsurprising that a taxonomy specifically adapted to a COVID-19 vaccination decision context differs substantially from previous ones. Furthermore, these factors, which are reflected in our data, contribute to a decision environment where people who are normally vaccine acceptant might refuse or, at the very least, delay a COVID-19 vaccine.

### Implications of results

We believe our HUVD taxonomy provides a valuable etension of existing models to a COVID-19 context, with possible relevance to future pandemic contexts with high uncertainty vaccination decisions. Past research has shown that public health messaging that targets individuals in a personalized and stage-specific manner can improve a variety of health outcomes, such as smoking cessation [39] and vaccine uptake [40, 41]. However, successful tailoring of messages relies on having a clear understanding of the decision factors present in the target audience. Our proposed taxonomy provides a similar tool to what has been created in the past, but specific to COVID-19 vaccine hesitancy and potentially other contexts with high uncertainty vaccination decisions. Such a tool can be used to bolster existing public health strategies around COVID-19 but could also be informative for future research into factors related to vaccine hesitancy in a pandemic context where high uncertainty vaccination decisions take place.

### Limitations

One major limitation of this work is that our taxonomy, while informed by qualitative data, has not yet been empirically tested in an applied setting. In addition, one must keep in mind that COVID-19 presented a unique context that may or may not generalize to other types of high uncertainty vaccination decisions–whether in a future pandemic or in other contexts. Nonetheless our taxonomy provides a useful starting point for developing public health messaging hypotheses to be tested in the particular context where they are to be applied. Additionally, while public health messaging designers can use our taxonomy to inform hypotheses they have about the types of messaging that may be most effective, they should not assume that a particular source of hesitancy is relevant without further empirical evidence.

A further limitation is the use of purposive sampling, whereby potential participants were selected based on their self-identified vaccination status and attitudes. While this approach allows us to recruit a relevant sample, it introduces the potential for bias due to self-selection by people with stronger opinions who are interested in participating in focus groups therefore potentially excluding more moderate voices. Related to this, focus group dynamics can potentially introduce bias due to groupthink. While the focus group moderators have extensive training and experience conducting qualitative research, this potential source of bias remains. A further limitation related to the focus groups is that because of the unique situation and limited sample size, we were unable to take steps to ensure data saturation.

A limitation related to the analysis of the data is the background of the qualitative data coder. While this coder has an educational and professional background in public health and qualitative analysis and vaccine hesitancy more specifically, any coder will bring a certain level of bias which must be recognized. Given the coder’s background, this bias could plausibly result in some level of confirmation bias–which we have attempted to address by carefully reviewing the raw data provided in the S2 Appendix.

Another limitation of the present study is the skew toward female and white participants in our sample. In an effort to select a sample that contains diversity in geography, educational status, income, and age, as well as a large representation of Indigenous communities, an unintended outcome was a final sample that contained an over-representation of women and an under-representation of ethnic groups other than persons of Indigenous ancestry. Finally, as noted in the Methods section, none of the male participants had children. Given that a significant amount of vaccine hesitancy attitudes and vaccination decision experiences happen in the context of childhood vaccines, this may have limited the representativeness of our sample.

### Recommendations for future research

Future work looking to expand on this taxonomy should focus on understanding how generalizable it is to populations in other jurisdictions and vaccine contexts. It should also make efforts to understand how our findings may be generalized to future pandemic contexts where high uncertainty vaccination decisions are inherent. In addition, we believe that work attempting to connect the taxonomy proposed here with empirical data from communication strategies will be useful in helping us understand the extent to which the taxonomy might be relevant in a real-world context, such as public health message design.

## Supporting information

S1 Appendix Definitions of themes identified in the qualitative data. (DOCX)

S2 Appendix Results of MAXQDA analysis. (DOCX)

S3 Appendix Focus group guide. (DOCX)
