# Supplementary material for: Navigating the uncertainty: A novel taxonomy of vaccine hesitancy in the context of COVID-19
Source: PLoS One. 2023 Dec 21;18(12):e0295912. doi: 10.1371/journal.pone.0295912 (PMC10734916; doi:10.1371/journal.pone.0295912)
Supplement: S1 Appendix — (DOCX) [file pone.0295912.s001.docx]

# S1 Appendix: Definitions of themes identified in the qualitative data

1. **Personal**
   1. *Value-based: This theme arises when individuals have different values or beliefs that may influence their vaccination decision. This could include religious or cultural beliefs, personal values, or ideological positions.*
      1. *Religion:* Respondents believe that getting COVID-19 vaccines may violate their religious regulations or beliefs.
      2. *Culture:* Respondents' uncertainty about COVID-19 vaccines is triggered by a mix of broader cultural and attitudinal factors, including anti-authoritarian worldviews, conspiracy ideation, or an alignment with alternative/complementary or holistic health.
      3. *Political Compass:* Respondents regard the COVID-19 vaccination campaign as highly politicized and question vaccines due to potential political ties.
      4. *Ethical Consideration:* Respondents worry that they may crowd out prioritized populations' vaccine supply.
      5. *Autonomy:* Individuals worry about having the freedom to make their own decisions regarding vaccination.
   2. *Trust-based: This theme refers to a lack of trust in the institutions, individuals, or processes involved in the development, approval, and distribution of the vaccines. This could include mistrust of the pharmaceutical industry, healthcare providers, or government officials.*
      1. *Trust in the medical profession:* Respondents regard medical doctors as unreliable information sources for making vaccination decisions.
      2. *Trust in the medical caretaker:* Respondents regard medical caretakers (e.g., a nurse) as unreliable sources of information for making vaccination decisions.
      3. *Trust in Government:* Respondents' trust level in government alters their decision to vaccinate according to governmental recommendations or responses.
      4. *Trust in Pharmaceutical Companies:* Respondents mistrust pharmaceutical companies or manufacturers.
      5. *Trust in Science:* Respondents believe that scientists are biased and that the scientific method is just a veil for pushing a political agenda.
      6. *Trust in Media:* Respondents are unsure which media provide reliable information on COVID-19.
   3. *Social: This theme arises from the influence of others, including friends, family, or community members. This could include social pressure to avoid vaccination, or the influence of misinformation or conspiracy theories shared by others.*
      1. *Pressure from Close Community:* Attitudes of respondents' close social circle impact their view on COVID-19 vaccines. The close social circle includes friends and family, with whom respondents have a close relationship.
      2. *Pressure for Broad Community:* Attitudes of respondents' broad community impact their view on COVID-19 vaccines. “Community” refers to people respondents may not know in person or have close relationships with but with whom they share identities.
      3. *Pressure from Society:* Respondents feel that unvaccinated populations are discriminated against and stigmatized as selfish or “anti-vax.”
   4. *Anecdotal: This theme refers to personal experiences or anecdotes that may have influenced attitudes toward vaccination. This could include negative experiences with vaccines in the past, or anecdotes shared by others about their experiences with vaccines.*
      1. *Personal Experience:* Respondents' uncertainty is triggered by their bad experiences with vaccines in the past.
      2. *Experience of close others with vaccines:* Respondents' uncertainty is triggered by bad experience of close others with vaccines.
      3. *Hearsay:* Extensive anecdotes around COVID-19 vaccination circulating on social media that trigger respondents’ uncertainty on vaccines.
2. **Scientific**
   1. *Risk-based: This theme relates to perceptions of risk associated with getting vaccinated. This could include concerns about the potential side effects of the vaccines, or fear of contracting COVID-19 from the vaccine itself.*
      1. *Short-term side effects:* Respondents are concerned about short-term side-effects (e.g., pain in the arm, headache, etc.).
      2. *Long-term side effects:* Respondents are concerned that the COVID-19 vaccines are untested and could cause unknown side effects in the long term.
      3. *Risk of serious symptoms or death due to COVID-19:* Respondents worry about the possibility of severe illness or death caused by COVID-19.
   2. *Knowledge-based*: *This theme refers to a lack of knowledge or understanding about the safety, effectiveness, or other aspects of the vaccines. This could include uncertainty about the scientific evidence supporting the vaccines, or confusion about how the vaccines work.*
      1. *Effectiveness of vaccine:* Respondents are unsure about the effectiveness of the COVID-19 vaccine in preventing COVID-19 infection or reducing symptom severity.
      2. *Understanding of vaccine function:* Respondents misunderstand the mechanism of action of COVID-19 vaccines.
      3. *Belief that natural immunity is better:* Respondents prefer to rely on natural immunity rather than artificial interventions, like vaccination, in preventing COVID-19.
      4. *Belief in alternative medicine:* Respondents take alternative medicine as a substitute for vaccination.
      5. *Information Overload:* Respondents are exposed to mixed or inconsistent messages asserting positive and negative views of COVID-19 vaccines, which cause confusion about which information is correct and which information sources are reliable.
   3. *Environment: This theme reflects the probabilistic nature of the world, the imperfection of measurements, etc.*
      1. *Possible evolution of the vaccine:* Respondents worry about the evolution of the vaccine (e.g., that it will be a monthly forced vaccination).
      2. *Flawed measurement of the vaccine:* Respondents worry that the methods to measure the effectiveness of the vaccine are flawed.
      3. *Possible evolution of the disease:* Respondents worry that the evolution of COVID-19 will reduce the relevance and effectiveness of the vaccine.
      4. *Flawed measurement of the disease:* Respondents worry that the detection strategies for COVID-19 do not work as designed.
3. **Practical**
   1. *System of Care: This theme relates to the processes or systems in place for administering the vaccines. This could include concerns about access to the vaccines, difficulties in scheduling appointments, or confusion about the vaccination process.*
      1. *Concern about booking:* Respondents are concerned about friction during the process of booking COVID-19 vaccines.
      2. *Vaccine supply:* Respondents have not received the vaccine due to limited vaccine supply.
      3. *Being out of commission:* Respondents worry that vaccine side effects will make them unable to meet the responsibilities.
      4. *Loss of access to public services:* Respondents worry that not being vaccinated may cause them to lose access to certain public services.
      5. *Time cost:* Respondents worry about the time required (for example, away from work) to get vaccinated.
      6. *Vaccine cost:* Respondents worry that they cannot afford the COVID-19 vaccine.
      7. *Transportation cost:* Respondents worry that they need to pay extra fees to get vaccinated (i.e., transportation cost).
