# Supplementary material for: Navigating the uncertainty: A novel taxonomy of vaccine hesitancy in the context of COVID-19
Source: PLoS One. 2023 Dec 21;18(12):e0295912. doi: 10.1371/journal.pone.0295912 (PMC10734916; doi:10.1371/journal.pone.0295912)
Supplement: S2 Appendix — (DOCX) [file pone.0295912.s002.docx]

# S2 Appendix: Results of MAXQDA Analysis

### Personal

We found that despite being exposed to similar information and changing circumstances, participants made different choices based on a variety of factors, including their level of trust in different information sources, the influence of their social circles, and their own values and beliefs.

#### *Value-based*

For most participants, the decision to vaccinate or not is related to a number of broader cultural and attitudinal factors. For example, Lorry and Elise observed the highly politicized trend of COVID-19 vaccine campaigns. According to Elise, “just in Alberta, it’s become very politicized… because of support and losing the future election is on the line.” Lorry also commented that “COVID vaccines are now up there with religion and politics for things you don't want to talk about with people that you don't really know.” While none of the participants took vaccination decisions as strong indicators of individuals’ political positions, we found that participants’ distrust of  politicians affected their decisions. Janet’s trust in potential serve outcomes of COVID-19 collapsed once she found “political gains” and “financial ties” between medical professionals and big foundations.

we've heard about the restaurants [banning vaccinated individuals], but are they going to start allowing… children to go to school without that vaccine or because they have religious or political affili… it as opposed to someone who's just medically has a problem with it? So… what's going to happen? Are the schools going to be allowed to say, sorry, little Timmy can't come to school if you don't vaccinate him. (Patrick, 53-year-old, vaccinated)

In addition to political ideologies, participants also linked their vaccination decisions to the freedom of an individual to make their own choices. Five unvaccinated participants (Eleanor, Ethan, Molly, Charles, and Janet) discussed feeling forced or experiencing limited autonomy because of vaccination campaigns that included pro-vaccine messages and vaccine passports, causing resistance toward vaccination. Ethan and Molly expressed dissatisfaction with the idea of a vaccine passport, stating that such restrictions would make him less likely to get vaccinated.

But… how they were restricting… access to certain things, and… if we don't have the vaccine… which I really didn't like… for me that… makes me… less likely to get the vaccine, because… it's not really acceptable to me. (Ethan, 26-year-old, unvaccinated)

The vaccine passport, that is something that concerns me more than motivates me. I can go without going to a restaurant, it's not going to break me. I can…handle that, if i'm not comfortable with it, giving me ultimatums… maybe it's my stubborn side, maybe it's my Dutch side, but it's going to make me push back… it just feels too forced, and for that reason I stand by my "I need more time and I need to make a decision that's best for me and that i'm comfortable with and have the freedom to choose. (Molly, 26-year-old, unvaccinated)

Words like “condescending”, “passive-aggressive”, and “pushy and exclusive” were frequently used by participants when discussing messages about COVID-19 vaccines, which evoked feelings of exclusion and stigmatization. Janet felt as if she was being "exterminated" from society for not getting vaccinated. "When you press people, it's going to make them push back 10 times as hard," she stated. Such a sentiment was shared by Molly, who discussed how her attitudes toward vaccines were shifted by pro-vaccine messages from the government and social media.

From the start, I was … pro-vaccine. But … it was actually when it became such a push [and] … I started to feel like I didn’t have a choice or I was going to be penalized [for not being vaccinated that I became more hesitant] … I feel like they’re trying to be neutral, … but I do find that it’s fairly … passive aggressive … The message that comes across is: ‘don’t be selfish, vaccinate to save others.’ Or ‘vaccinate to save yourself’…I don’t like that … It’s a message like I was cornered, … not feeling like I have a choice …that honestly makes me push back against [getting vaccinated] … I just don’t feel like I can make a decision that’s best for me. And I’m not trying to be selfish, and that’s where I get frustrated with … the media messages … I want to make the best decision for me, and I don’t think I’m being selfish by choosing, at this point, not to get the vaccine.  (Molly, 26-year-old, unvaccinated)

Nine participants voiced out that people "should be taken on [their] free will [to make vaccination decisions]," and "it's everyone's choice what they feel is best for them." Esther, who had already been vaccinated, stated that "it's everybody's decision, whether they want to get [vaccinated] or not…[she wishes] everybody would. It might bring cases down more, less death, less sick people. But it's up to everybody." William called for accepting people for who they are and their decision to vaccinate or not. "It's your decision…we should accept people for who they are [and] embrace that."

#### *Trust-based*

Throughout the focus group discussions, participants expressed varied views on the trustworthiness of medical professionals, health providers, the pharmaceutical industry, and government officials in the development, approval, and distribution of COVID-19 vaccines. Trust toward these entities influenced the information sources they seemed to rely on.

Participants who were vaccinated or intended to get vaccines in the future cited medical professionals such as Dr. Anthony Fauci as trustworthy sources of COVID-related information. For example, Billy, who was not vaccinated yet but planned to receive one eventually, took Dr. Fauci’s suggestions as “the gold standard” during his decision-making process.

Dr. Fauchi is basically the gold standard for you know scientists who know, and so I pretty much relied on him… Dr. Fauci and the experts that go on TV. They've also clarified the side effects (Billy, 64-year-old, unvaccinated)

Elise also expressed her preference for having medical experts deliver COVID press releases.

I have a lot of friends who work for Alberta Health Services, so mostly i've been trying to get more information from them, as well as doctors and government websites… I would like if we had more medical experts, or at least working infectious disease experts, to be the ones doing the COVID press releases right now. (Elise, 28-year-old, vaccinated)

Not everyone shared the same confidence in medical professionals. Patrick found conflicting views from medical professionals, prompting him to turn to independent news companies that compared different sources of information to ensure the reliability of the COVID-related information he received.

I listen to different health sites and all that, but I started finding a lot of conflict in what they were saying, including Dr. Fauci… He was one day he was saying it wasn't caused in the lab, you know… the official sites weren't giving the right news. So I actually started turning to alternative news, independent news companies and found that… they would compare notes from all the different sources. And then through a company called News Guard, they would be able to distinguish what was real and what was [not real]... it gave me a little bit of a better understanding rather than only taking it from one source. (Brooke, 53-year-old, vaccinated)

Trust in medical professionals also made some participants more susceptible to misinformation and fake news. For instance, William cited a statement made by Dr.Michael Eaton in an interview with Life Site News as “proof” of COVID-19 safety and noted that “we are dealing with a conspiracy which is beyond the carnage.”

from April 7 2021, Life Site News, Dr. Michael Eaton, Pfizer's former Vice President chief scientist for allergy and respiratory, who spent 32 years in the industry leading new medicines research and retired from the pharma-school giants with the most senior research position in his field, spoke with Life Site News on an interview. He addressed the demonstratively false propaganda from governments in response to COVID 19, including the lie quote of dangerous variants, the totalitarian potential for vaccine passports and the strong possibility we are dealing with a conspiracy which is beyond the carnage. (William, 44-year-old, unvaccinated)

When it comes to pharmaceutical companies, the landscape was completely different. Janet voiced out that there is insufficient liability for vaccine manufacturers, which she argued should be in place to encourage safer vaccine development.

I don't think that there's enough liability for - well there's no liability for the manufacturers, right now, and I think there needs to be, because I think that if there was it would push them to be safer. Because when you're being liable for the lawsuits instead of the tax payers, then you're kind of pushed to make them as safe as they could possibly be, regardless of an extra 10 cents a cost provide… I think that they're [vaccines] a good concept, a good design, but they're not being pushed to be as safe as they could be because the manufacturers aren't being held liable, not in Canada, not in the States. (Janet, 26-year-old, unvaccinated)

Participants' medical caretakers played a key role in their vaccination decisions. Unvaccinated participants such as Melanie chose not to vaccinate based on the recommendations of specialists. After speaking with two oncologists, Melanie and her doctors agreed that while she was at high risk of contracting COVID, she faced an even higher risk of getting sick or worse from the vaccine due to her medical conditions. Consequently, she decided not to get vaccinated, opting to follow the advice of her oncologists over her family doctor.

With the talk that I've had with two oncologists, we just agreed that I'm a high risk of getting COVID, but I'm a higher risk of getting sick or worse from the shots, so [I decided to not get vaccinated]... My family doctor said it would be okay, it would be safe. My two oncologists said no don't because of the chance of a blood clot, so I went with the odds of the two oncologists agreeing because they're more involved in my health issue than the family doctor. (Melanie, 52-year-old, unvaccinated)

Medical caretakers' opinions also contributed to the uncertainty in Brooke's decision about getting vaccinated. Her doctor informed her that, given her health conditions, she could experience a severe reaction to the vaccine. This information left her unsure about whether to proceed with vaccination.

my doctor alone told me with my conditions that there's a chance that I would have a bad reaction to the shot. My doctor wasn't even 100% sure on what of side effect would happen to me because of having a certain arthritises I have and scoliosis and my lungs being filled with liquid already, he wasn't sure what would have happened if I took the shot, muscle or anything wise, so he said, you can take it. But there, you will have a bad reaction, so that kind of put me on the fence. (Brooke, 36-year-old, unvaccinated)

In contrast, some vaccinated participants made their decision to get vaccinated based on their medical caretakers' recommendations, even if they were initially hesitant. For instance, Esther mentioned that the Arthritis Society played a crucial role in her decision to get vaccinated. Their outreach and encouragement to get the vaccine for her safety convinced her to proceed.

I didn't feel I had enough [information to make vaccination decisions]... I got arthritis, severe osteoarthritis, so I had the Arthritis Society, I've signed up to them and they've been sending me emails about messages… because I wasn't sure if I was going to get the vaccine or not, but they recommended me getting it… because they, [the Arthritis Society] said because I have osteoarthritis, I've had two knee replacements in nine years. They're like, You should get the vaccine for safety, for your safety and well-being. So I got it.

Similarly, Joy, a 49-year-old woman who was initially hesitant to get vaccinated, changed her mind after her pharmacist reassured her that her risk of side effects was manageable.

I heard that people were dying getting the shot, after they got the shot that they were dying. So I was quite scared to get it… I've had allergies and like anaphylactic to stuff before, so I was really worried about getting it, because in the beginning they said anyone with anaphylactic couldn't get the shot. So when [my pharmacist] talked to me about it and he said, it's OK now,they did studies, and you should be OK. So I finally did it. (Joy, 49-year-old, vaccinated)

During the focus group discussions, we observed that participants generally showed trust towards medical professionals and caretakers. However, it became apparent that suggestions from medical caretakers carried more weight and had a greater influence on the participants' attitudes and decisions. This could be attributed to the perception that medical caretakers possessed a deeper understanding of their individual medical condition and were therefore better suited to make decisions in their best interests.

Nevertheless, trust towards medical professionals and caretakers can be undermined when there is a perceived political or financial conflict of interest. For example, Janet shared how her attitude changed after discovering financial ties between the Gates Foundation and Neil Ferguson from the Imperial College of London: “for the first two or three weeks, I mean, I was very serious about it… and I find out Bill Gates funded those too. So it’s just like, how can you trust, you know when there’s all of these financial ties, political gains.” Later, she expressed concern about censorship from the government to doctors “that have spoken out” and were “sharing valid things.”

So those kinds of things just are raising major red flags for me, and I feel like from the doctors that have spoken out, Dr. Byron Biddle, Dr. Christina Parks, Dr. Sunetra Gupta… they're… sharing valid things and they're being censored [from government], and that should raise red flags for everybody. (Janet, 26-year-old, unvaccinated)

Janet was not the only participant who became hesitant when they perceived political intervention.

I think science in general is what I trust, but who I don't trust is politicians For instance, Toronto has a great medical health doctor… they're being allowed to give us great information, but the politicians are trying to spin it. That's where I have a problem with it. (Brooke, 53-year-old, vaccinated)

Other participants questioned politicization trends around COVID-19. Elise expressed her concern that “it's become very politicized- a lot of stuff is being done right now, because of support and losing the future election is on the line.” and stated that she would like “more medical experts, or at least working infectious disease experts, to be the ones doing the COVID press releases right now.”

specifically my issue is just related to our provincial government, I would like if we had more medical experts, or at least working infectious disease experts, to be the ones doing the COVID press releases right now. Just in Alberta, it's it's become very politicized… because of support and losing the future election is on the line. (Elise, 28-year-old, vaccinated)

Other participants maintained their faith in the government. Six participants cited governmental websites like Health Canada as one of their main information sources. And two participants cited approval from Food and Drug Administration (FDA) as supporting evidence during their decision-making process. But the impact of FDA approval is limited. Billy stated that FDA approval did not “help in at all fears” of COVID-19 vaccines’ side effects. Molly expressed her “wanting an FDA approval” of COVID-19 vaccines but her story about chicken pox vaccines implied that FDA approval did not determine whether or not she trusts vaccines.

The vaccines [my kids] have had gone through the normal FDA approval before I would allow them to have it. But that being said, I struggled with say giving the chickenpox vaccine. And part of the reason of that, and I did end up giving it to to my son and my daughter, because I looked into it and I found that the risk of side effects were so small, and it had been FDA approved, and I looked into that. But the main reason I was hesitant on giving it to them or having them get it… was because chicken pox isn't a deadly disease. (Molly, 26-year-old, unvaccinated)

In addition to governmental information sources, participants use traditional media such as CTV, CBC and CNN, search engines like Google and Yahoo, and social media platforms like Facebook and Twitter. Both vaccinated and unvaccinated participants were exposed to multiple information sources. Nevertheless, vaccinated participants (Julie, Clara, and Lorry) seemed to place higher trust in governmental or mainstream media sources, such as CTV and Health Canada, while unvaccinated participants (Eleanor, Ethan, Molly in group C) appeared to place greater trust in social media platforms. This contrast becomes more apparent when considering comments from Lorry, a 30-year-old vaccinated participant, and Molly, a 26-year-old unvaccinated participant:

So I get the majority of my news from CBC and different other like local news, CTV… and then my doctor sends out like newsletters with COVID updates, usually once a month, and then I as well follow City of Ottawa, puts out a lot of sort of information through social media even Instagram. So I follow all those news sources that I rely on for reputable information and… I see a lot on social media and a lot through word of mouth and through friends, but… I take that with a grain of salt, I don't really believe much of what I see on Twitter, so my main news sources would be CBC, CTV usually. (Lorry, 30-year-old, vaccinated)

I… would say Facebook. I spend a lot of time kind of scrolling, which I don't like… the news sources just pop up. Now that being said, they still are… reputable sources such as… CNN and CBC, CTV. Putting out articles that other people have shared, but I don't particularly like reach out and look for information and it's kind of what pops up. (Molly, 26-year-old, unvaccinated)

The level of consistency in released information may contribute to their different vaccination choices. While information overload added uncertainty for both vaccinated and unvaccinated participants, unvaccinated participants, who considered social media as their main information source, faced a more inconsistent and diverse range of information.

There's a lot of mixed messages on social media that I've seen… but you don't really know... if it's credible, like you always have that wonder like is it right, is it not right… it plants the seed of doubt… I have read and absorbed and heard a lot of [horrible stories about getting vaccinated] so like I said it plants the seed of doubt in there, like it makes you wonder… Cause some of those things might actually be real. (Eleanor, 43-year-old, unvaccinated)

#### *Social*

Social uncertainty — arising from the influence of friends, family, or community members — emerged as a key theme. Pressure from participants’ close community, such as friends and family members, directly affected participants’ perceptions of COVID-19 vaccines. Julie, Joy and Brianna made clear statements that they trust their families’ opinions the most on COVID-related issues.

Close community pressure seemed to have a significant impact on participants' vaccination decisions, even in cases where participants did not explicitly state it. Among vaccinated participants, we heard a number of stories about how close social circles encouraged them to get vaccinated or how they themselves encouraged others to get vaccinated. When asked how their social circle reacted to her decision to get vaccinated, Esther mentioned that her parents “were happy” about it. She later shared her experience persuading her oldest daughter to get vaccinated, citing the potential exposure to COVID-19 due to their jobs.

she refused to get vaccinated, but I said to her, look, I said, your father and I both work where we are in contact with people 24/7, because I work at a fast food restaurant, my husband works at a lumberyard, he's a truck driver. And I said, we don't know if we're if we're going to end up coming in contact with someone that's not vaccinated, that has it. So she finally got vaccinated. (Esther, 60-year-old, vaccinated)

Patrick took a different approach when persuading his nephew to get vaccinated, highlighting the benefits of being vaccinated for travel purposes.

I actually had my nephew say he was worried about the vaccine and all that, and the way that we finally convince him to get it is because he loves to travel, he was supposed to go to Finland sometime this winter and it's like, hey, you can't go if you're not vaccinated. And so then he went and got vaccinated. (My sister had , 53-year-old, vaccinated)

Some participants felt pressure from their close communities, not necessarily due to explicit attitudes, but rather due to their desire to protect loved ones. For example, Clara said: “I was scared of my daughter and my family because my dad has a bad like breathing problems, so like I was scared of she’s going to get it or, you know, because it wipes out family sometimes.” Esther shared a similar feeling of “being scared to get it [COVID-19]” because “it’s not much [about] me, it’s the fact that if I get it, I can’t see my dad for a while because of him being ill.”

Unvaccinated participants responded quite differently. Their close friend circles remained neutral or shared hesitancy about vaccination. Ethan noted that he received different opinions from his friends.

Some… really didn't have much of an opinion, some didn't care, some of them were like the same like me… they weren't planning to get the vaccine… some of my other friends were… encouraging me to get it, and other ones… were… really pushing me to to get it… a lot of different opinions.(Ethan, 26-year-old, unvaccinated)

Molly, on the other hand, found her support from her close friend circles, which also shared her hesitancy towards getting vaccinated.

I have a friend, a circle of friends that are hesitant about the vaccine. And we're all coming from the same place of if you feel comfortable getting vaccinated, please do, do what you feel comfortable with, but please respect our decision not to at this time. (Molly, 26-year-old, unvaccinated)

In some cases, the pressure to get vaccinated may come from wider communities that participants are part of. Molly mentioned that her father felt “excluded” and left “out of everything” because “he’s not vaccinated”.

he can't get vaccinated and he's feeling like… completely out of every thing now because he's not vaccinated… it's like horrible because he's lived here for seventy-six years. And he said now he doesn't feel like he belongs at all like the government's totally made him feel like he's a nobody, he said… it's not like necessary to me because and it's not like… he's against vaccines. He gets the flu shot, he gets every other vaccine except for this one. (Molly, 26-year-old, unvaccinated)

Molly was not the only one who noted this kind of exclusionary experience. Six participants shared that unvaccinated individuals are discriminated against and stigmatized by society, being labeled as “selfish” or “anti-vaxxers.” Patrick said: “They’re all getting labeled as Karens because… of the old anti-vaxxer thing. ” Similarly, Brianna observed that “I haven't really heard anything about people not getting it and other people saying things about them, except for the self-being selfish or something like that.” Julie, Brianna, Molly, Esther, Ethan, and Janet stated that it is “unfair” to label unvaccinated individuals as “selfish ” as “it’s an own personal choice” and people “should take on their own free will.”

Participants reported that social pressure is often reinforced by targeted COVID-19 vaccine campaigns that contain messaging such as “don't be selfish, vaccinate to save others.” Unvaccinated participants further explained that the messaging made them feel shamed and separated them socially and morally from the unvaccinated population. As Janet, a 26-year-old unvaccinated participant stated, the messaging implies that “[The messaging] implied [that] … you’re not a good person…you’re not protecting your family, maybe you even want them dead [if you choose not to get vaccinated]…it’s not ‘we’re in this together’ messaging.” Eleanor, another unvaccinated participant, shared that “I find the ads are not only pushing it but they're making people who don't choose it feel like they're outcasts and there are people who have had a lot of bad backwash from that i've heard of people actually being bullied because they don't get it.”

The feelings of exclusion could be exacerbated by the concept of the vaccine passport. Participants expressed concerns that unvaccinated individuals may be banned from restaurants, events, or even school, leading to feelings of isolation and alienation. Ethan shared his experience of being rejected by a barbecue group because he was not vaccinated. Molly commented that the vaccine passport “concerns me more than it motivates me.” Patrick viewed the idea of banning unvaccinated individuals from restaurants or travel as a “sort of totalitarian thing” and questioned: “Are they going to start allowing… children to go to school without that vaccine or because they have religious or political affiliations… it as opposed to someone who's just medically has a problem with it?”

#### *Anecdotal*

We found that participants' personal experiences with COVID-19 vaccines or the virus itself could greatly influence their vaccination decisions. For instance, Joy, a participant in our research, experienced uncertainty about COVID-19 vaccines due to her negative reaction to the Moderna vaccine. She felt unwell for two months after receiving her second dose, which led her to question the efficacy of COVID-19 vaccines compared to other vaccines, at least for her personally.

I never had any reactions, and after I got my two shots of Moderna, I've been like so sick. And it's been like two months since I had my second shot, and I'm still feeling horrible. So for that reason, I don't really think they're as good as the other vaccines, at least for me personally. (Joy, 49-year-old, vaccinated)

On the other hand, Brianna's experience with negative effects resulting from COVID-19 drove her to get vaccinated. She mentioned that she still deals with lingering side effects, such as the loss of smell and taste, which she found to be a terrible ordeal. Brianna's experience with the virus and its lasting impact convinced her of the importance of getting vaccinated to prevent further complications.

I have more side effects that I'm still dealing with from having COVID-19… that was like crazy. And I still find that at different times that I can't smell or I can't taste… Because it was a terrible… thing. And if people only knew how terrible it is, and then there's still the lasting effects from it, too. That's what - that's what got me [vaccinated]. (Brianna, 63-year-old, vaccinated)

Among unvaccinated participants, stories about negative experiences of having extreme side effects of COVID-19 vaccines were prevalent. Janet shared her friend's experience of having a collapsed lung and being temporarily paralyzed after getting vaccinated. “Very serious injuries…not just minor injuries” she emphasized, “that scares [her], especially in the younger population where COVID doesn’t really affect too much.”

because I know people with injuries to the AstraZeneca and the Pfizer vaccine. Very serious injuries, as I stated before, not just minor injuries. Not life threatening either, but I would still consider a collapsed lung pretty serious and the temporary paralysis of the other boy happened on the road, so you know he could have been in a very serious car accident, he had to use his chin to pull himself over because he lost feeling from the neck down. So that scares me, especially in the younger population… where COVID doesn't really affect too much. (Janet, 26-year-old, unvaccinated)

Similarly, Molly and Patrick heard stories from their families and neighbors about adverse side effects from the vaccines, which contributed to their uncertainty.

My sister had terrible side effects, vomiting for two days just you know where she needed somebody to come and take care of her and that was really worrisome. (Molly, 26-year-old, unvaccinated)

I actually had a neighbor who he did, he had the Pfizer vaccine, and he had really bad side effects for a day or two after, and you know it made me kind of pause. (Brooke, 53-year-old, vaccinated)

Clara was also hesitant about vaccination after her friend contracted COVID-19 despite being vaccinated.

I was… iffy about it, because my one friend, he got… the needle. And then he still got COVID. So like, I didn't know, maybe he got COVID from… the shot. (Clara, 26-year-old, vaccinated)

On the other hand, Ethan, who remains unvaccinated, mentioned that his mother and friends experienced no side effects after vaccination, which may contribute to his intention to get vaccinated in the future.

I had like like my mother, for example, and another… friend and they had the vaccine and they both said that they didn't have any like side effects (Ethan, 26-year-old, unvaccinated) ,

The influence of the word-of-mouth, even from unknown individuals, shaped participants' attitudes towards COVID-19 vaccines. Our conversations with Patrick gave us insights into how participants’ attitudes change when they are exposed to different anecdotes. In the focus group, Patrick initially shared his experience talking with “one of the guinea pigs”, who “tried seven different vaccines … And… had no side effects to any of them.” This experience made him more certain about vaccine safety, as he noted: “there's got to be some safety there.” He ended up getting Moderna shots, however, he later mentioned that his neighbor’s severe adverse reactions to a Pfizer vaccine made him pause during the decision-making process.

Anti-vaccination campaigns, on social media like Facebook and Twitter, also affected participants. Participants in Group A reported that they found more “horror stories” than “good information about the vaccine” on social media. Lorry from Group C added that “the people who are the loudest on social media tend to be the people who are really against the vaccine and want everybody to know it on [her] Facebook.” Even though she “[doesn’t] want to get into those types of conversations”, she had “notched [them] down in [her] confidence levels [because she had been] … seeing so many anti-vaxxers … [on] Facebook … [that] sort of accidentally [had] gotten into [her] head a little bit.” Participants like Eleanor acknowledged the lack of credibility of some stories posted on social media but admitted that “[those horror stories about vaccines plant] the seed of doubt in [his mind]... cause some of those things might actually be real.”

### Scientific

Scientific uncertainty  was another category of themes that was prevalent in our focus group discussions.

#### *Environment*

Participants expressed a pervasive sense of uncertainty about COVID-19 and COVID-19 vaccines. Many of their concerns were driven by the unpredictable nature of the disease and its potential evolution which left some feeling uneasy even if they had been vaccinated. Elise and Patrick, for instance, shared concerns about new variants and the potential for more contagious strains to emerge:

"Now… we have this fourth variant, or this one new variant it's mutating, and that's…really scary… and they're saying that this new variant is more contagious but less harmful… that doesn't give me comfort." (Patrick, 53-year-old, unvaccinated)

"I'm worried about how big COVID's going to get with the new variants; I'm worried about our society as a whole right now." (Elise, 28-year-old, vaccinated)

Some participants expressed skepticism and doubt about the true nature of COVID-19, suggesting the possibility of flawed measurement or flawed understanding of the disease by experts. Molly, for example, questioned why COVID-19 is considered more important than other viruses:

"For this virus to be so important over others… it does make me concerned… why the government needs to have control over this situation." (Molly, 26-year-old, unvaccinated)

Others noted conspiracy theories around the non-existence or exaggerated impact of COVID-19. Julie shared her brother’s view on COVID-19:

"It's with my brother because he doesn't want to get [the vaccine]... he thinks it's a conspiracy, and… it's like the flu." (Julie, 58-year-old, vaccinated)

Such conspiracy theories were prevalent during the pandemic, as Patrick, Billy, Brianna, and Lorry shared their experiences of being exposed to such information. Lorry, for example, shared that she had “notched [them] down in [her] confidence levels [because she had been] … seeing so many anti-vaxxers … [on] Facebook … [that] sort of accidentally [had] gotten into [her] head a little bit.” Brianna shared a similar feeling, saying that she knows "it's an unknown thing" but that "no one really had this before...it was uncertain."

As our conversations continued, we found that uncertainty regarding the probabilistic nature of the disease extended to perceptions around COVID-19 vaccines. The changing nature of the vaccination development process and rollout plan triggered inconclusive arguments and questions around the effectiveness and side effects of vaccines. Some participants attributed the ambiguity to the relatively short research and development period. Six participants (Janet, Melanie, Eleanor, Brooke, Ethan, and Patrick) explicitly stated that they believe the vaccines had not been adequately tested before being rolled out. When asked about an acceptable trial period, participants expected "a couple of years," allowing time to test the vaccine on a diverse range of individuals with different conditions.

Interviewer: Anyone that mentioned the trial period is there, you know, there was there were some comments it wasn't tested long enough. what what comprises long enough, like what would be long enough?

Eleanor: I'd say at least a couple years or more, you know there wasn't even that. A few years. (Eleanor, 43-year-old, unvaccinated)

Melanie: I agree at least a couple years, because then at least they'd have had a chance to test it on a variety of different people with different conditions. (Melanie, 52-year-old, unvaccinated)

The prospect of regular booster shots further fueled skepticism. Brooke (unvaccinated) and Joy (vaccinated) both pointed out the ongoing booster campaign makes people more doubtful about its effectiveness. Brooke stated “keep bringing out new ones” is “the only reason” why she doesn’t trust COVID-19 vaccines. She questioned, “How reliable are the actual vaccines if we have to keep getting vaccinated every five months?”

The other reason I don't trust them is they have to keep bringing out new ones, and I've heard and read and talked to other people that they're talking about even after taking the third dose, that five to six months later you're gonna have to take another dose... How reliable is the actual vaccine, if we have to keep getting vaccinated every five months? (Brooke, 36-year-old, unvaccinated)

I think that maybe if they did some more studies on it and more information about the results… it was kind of more rushed, and I understand it's a pandemic and they had to rush, but if they maybe waited a bit longer instead of… started so soon, because even now they're saying now we need a third shot. And then people are saying we're going to need a shot every year. I just think… all these things are making people more doubtful about getting the vaccine and more hesitant. (Joy, 49-year-old, vaccinated)

During our conversations, we also discovered that participants were worried about the possibility of inaccurate assessment of the vaccine due to the relatively short research and development period. Although not explicitly stated, participants expressed uncertainty and concern that potential adverse consequences of vaccination had not been thoroughly tested.

Well, if one is saying that there's a chance of getting a blood clot. Well, how can they say that the other two are going to be okay from – are going to be okay and you're going to be okay from it, when they haven't been properly tested. (Melanie, 52-year-old, unvaccinated)

Yeah I agree it's like pushed out so fast and it's like okay, good luck with that, no we don't know a lot about it but good luck. (Heather, 68-year-old, unvaccinated)

I was… thinking that usually most vaccines or anything…cures for cancer or whatever, take a long time before you find out if they're safe or not. (Brianna, 63-year-old, vaccinated)

In focus groups, participants seemed to grapple with the question, "Is there anything else about COVID-19 that we don't yet know?"

#### *Knowledge-based*

The changing nature of the disease and its vaccines led to ambiguity and confusion in COVID-related knowledge. One common confusion was around the effectiveness of vaccines. Both Eleanor and Brooke expressed concerns regarding the vaccines' ability to prevent infection and the potential for increased side effects with each booster shot. Eleanor shared her confusion around the effectiveness of vaccines:

I was wondering a lot well how effective, is it... does  risk for certain side effects go up every time you get a booster you know… questions led to more questions, I just found that there wasn't enough clarity to it. (Eleanor, 43-year-old, unvaccinated)

Brooke raised concerns about the apparent contradiction between the vaccines' purported safety and the ongoing precautions taken by vaccinated individuals:

Actually, that makes a good point, everyone that's double vaccinated, makes it kind of confusing when the people who aren't vaccinated still need to wear masks and social distance. If the vaccine was so safe, why is everyone double vaccinated still wearing masks? (Brooke, 36-year-old, unvaccinated)

More participants were uncertain about vaccines’ efficacy in preventing transmission, durability over time, and for those who previously contracted the virus, while they widely held belief that vaccines can protect individuals to some extent.

Can we still spread it to others with the vaccination?... I don't know if it's true that you can still get COVID, if you get it. (Clara, 26-year-old, vaccinated)

is it still as strong as it was at protection, like right after? Or did it weaken? (Joy, 49-year-old, vaccinated)

This skepticism was further fueled by reports of vaccinated individuals contracting and spreading COVID-19. One participant, Joy, who had received the vaccine, expressed her skepticism as she personally did not know anyone who had contracted COVID-19 and had been extremely sick. Later on, Joy expressed her continued skepticism by stating “by getting the vaccine, I’m protecting myself, but even if I had the vaccine, I can still spread COVID to somebody who doesn’t.” This realization did not serve her motivation to protect her high-risk father from COVID-19.

it's stopping people from getting super sick… I haven't seen it personally, I also haven't had any friends that have gotten COVID and been extremely sick… I feel like there's a…gap of of knowledge and… I just want to wait and see… my father in law is sort of high risk, he's diabetic so he has all of his vaccines. And… he tried to say to me, you need to go get your vaccine so that you don't spread it to me. And… to me is… where i'm seeing a lot of misinformation, where i've been told that vaccine doesn't stop us from spreading it… it stops that individual from getting it, so by getting the vaccine i'm protecting myself, but even if I had the vaccine, I can still spread COVID to somebody who doesn't. (Joy, 49-year-old, vaccinated)

The confusion around vaccines’ efficacy implied participants’ lacking knowledge in how vaccines work. In our discussion, Clara used the word “iffy” to describe her feelings when her friends contracted COVID-19 despite being vaccinated. “Maybe he got COVID from the shot” Clara further shared, suggesting that she may not fully understand the pharmacological mechanism of the vaccine in the first place.

I was…iffy about it, because my one friend, he got the [vaccine]. And then he still got COVID. So…I didn't know, maybe he got COVID from… the shot. (Clara, 26-year-old, vaccinated)

Given the indeterminacy of vaccine outcomes, unvaccinated participants turned to alternative methods, such as strict self-quarantine, and relying on their own natural immunity to protect themselves. These beliefs in alternative methods and natural immunity may stem from risk perceptions on vaccines’ side effects, which we will discuss later in this article. Eleanor expressed her “biggest fear” about “not enough being known about long-term side effects” and her preference for a “natural approach”. Heather strongly agreed with Eleanor and added that “fear of long term (side effect)” and “so much unknown” about vaccines led her to “rely on my own immunity”. Participants, with beliefs in alternative methods, shared similar concerns. Melanie, recovering from her cancer, “cut people off” and told her friends “it’s probably better that we FaceTime or texts or call”, because her oncologists agreed that “I'm a high risk of getting COVID, but I'm a higher risk of getting sick or worse from the shots”. Similarly, Brooke adapted her daily routine to minimize exposure to the virus, such as relying on online grocery shopping and avoiding physical contact with people or objects.

Several unvaccinated participants expressed a preference for a "natural approach" to protect themselves, citing concerns about the vaccines' long-term side effects and a desire to rely on their own immune systems. Eleanor expressed her “biggest fear” about “not enough being known about long-term side effects” and her preference for a “natural approach”. Heather strongly agreed with Eleanor and added that “fear of long term (side effect)” and “so much unknown” about vaccines led her to “rely on my own immunity”.

I've heard that it can be really bad for people that have respiratory issues. I have, like I said I have asthma, I have very bad allergies, I've had breathing problems… I'm also worried about long term effects things that people don't know about yet… I tend to take a more natural approach to my health, I'm not on any medications or anything like that, it's all about just eating certain things, meditation, stuff like that, yes, and putting your mind in the right kind of focus. I just – I'm not a big person with doctors and stuff so I try to take a natural approach to things. (Melanie, 52-year-old, unvaccinated)

what they said was exactly what I could say… the fear of long term. So much is unknown about it. Side effects, everything like that…So I kind of look after my own health and rely on my own immunity.  (Heather, 68-year-old, unvaccinated)

Although most participants in our study reported obtaining their knowledge about the pandemic and vaccines from official government sources such as the Public Health Agency of Canada, they also sought information from alternative sources such as mainstream media, independent investigators, and anecdotal evidence from friends and social media. Exposed to an abundance of information, participants found information, which presented positive and negative views and experiences with COVID-19 vaccines, conflicting and hard to keep up with. For example, Ethan shared contrasting experiences with COVID vaccines from his friends and a taxi driver.

My mother… and another… friend… they had the vaccine and they both said that they didn't have any… side effects, but then…a taxi driver… said that he had like side effects after the second dose… so… I found that… they gave a lot of information. (Ethan, 26-year-old, unvaccinated) ,

Julie noted the fickle nature of information released.

 It was just, there's so much information out there about it. And each day it would sort of change. (Julie, 58-year-old, vaccinated)

This exposure to contracting perspectives led to confusion among the participants about which information is accurate and which sources are reliable. Charles, who is unvaccinated, described his experience over the past one and a half years as going “from uncertainty to confusion.” He explained, “we’ve been getting a lot of information from different sources. And we want to get the information from reliable sources, and to be sure that the information that we are getting is true… that is where the confusion comes in now because we don’t even know where I mean these sources that we should trust.”

everyone kind of expressed a bit of like confusion, or that there's like mixed messages, or that there's different information kind of out there which is maybe leading to a bit of kind of lack of confidence in the in the information that's available.

Janet: Yeah (Janet, 26-year-old, unvaccinated)

#### *Risk-based*

One of the most commonly discussed themes was related to participants’ risk perceptions of vaccines. First and second-hand stories of individuals experiencing severe side effects or even death after vaccination were prevalent. Joy and Esther both heard about “people were dying or people were having really bad side effects”.

Joy: Yes, I heard that people were dying getting the shot, after they got the shot that they were dying. So I was quite scared to get it. (Joy, 49-year-old, vaccinated)

Esther: I heard the same, people were dying or people were having really bad side effects. (Esther, 60-year-old, vaccinated)

 A neighbor of Patrick experienced “really bad side effects for a day or two after [getting Pfizer]” which made him “pause” vaccination decisions.

I actually had a neighbor who he did, he had the Pfizer vaccine, and he had really bad side effects for a day or two after, and you know it made me kind of pause. But then I actually found out I was getting the Moderna one, and I didn't have any side effects whatsoever. (Brooke, 53-year-old, vaccinated)

“if one is saying that there's a chance of getting a blood clot. Well, how can they say that the other two are going to be okay from – are going to be okay,” Melanie concluded. Their shared fear was reinforced by news that “AstraZeneca [has] already been taken off the market because of very serious side effects.”

For participants with pre-existing medical conditions, the risk of COVID-19 vaccines is even harder to manage because they are unsure how the vaccines will interact with their health issues or medication. Melanie noted that, according to his cardiologist, his blood disorder put him at a high risk of developing blood clots.

My thing was with my cardiologist… because, again, there are some serious side effects that like… myocarditus, inflammation of the heart muscle… if I get COVID-19 it would be worse so ... that's why [I decided not to get vaccinated]. (Melanie, 52-year-old, unvaccinated)

Eleanor also expressed concerns that vaccines could be harmful to people with respiratory issues.

I've heard that it can be really bad for people that have respiratory issues… I have asthma, I have very bad allergies, I've had breathing problems. (Eleanor, 43-year-old, unvaccinated)

Brooke was “on the fence” because her doctor “wasn’t even 100% sure of what side effect would happen to me because of having certain arthritis”. Later on, she asked, “if I was on a medication and if I take the COVID vaccine, is it going to counteract my other medication and cause whatever that was helping to fail?”

We observed similar ambiguity when it comes to long-term side effects. Eleanor was “worried about long-term effects things that people don't know about yet that can happen years down the road like what of that.”

My biggest fear about this whole thing is that… it was new it came out quickly and not enough is known about long term side effects. Everyone that's taking it… you're the test subject and… they don't really know what's going to come of this whole thing in the long run… I'm fearful of the long term effects it can have not, just immediate effects of just taking the vaccine right away… I've even heard things…that affects on women with their their fertility and menstrual problems and stuff, like I have heavy menstrual now, I mean I couldn't even imagine if I had that sort of side effect (Eleanor, 43-year-old, unvaccinated)

Charles’ friends “don't want to get the vaccine because of some of the information that they get, that if you get a vaccine you are going to, maybe after two years”.

some people, like some of my friends, they don't want to get the vaccine because of some of the information that they get, that if you get a vaccine… maybe after two years, you are going to die or something… they're saying that the government is using that to reduce the population. (Charles, 31-year-old, unvaccinated)

Risk perceptions surrounding COVID-19 vaccines co-exist with risk perceptions related to the disease itself. However, we observed noteworthy variances in risk perceptions among vaccinated and unvaccinated participants, which may lead to their divergent vaccination decisions.

Most unvaccinated participants considered themselves at low risk of severe symptoms or death related to COVID-19. Molly, who is 26, cited her demographic group as “considered very low risk” and mentioned that she lives in an area with minimal cases. William said he doesn’t “know anyone that's died from” COVID-19. Same as Janet, who knew “people with injuries to the AstraZeneca and the Pfizer vaccine” but didn’t see where COVID really “affects too much” the “younger population”.

On the contrary, we found that vaccinated participants are more concerned about potential risks from COVID-19 itself. “COVID would definitely kill me, so I chose to take it [COVID-19 vaccines],” said Billy, who at the same time had heart inflammation and could potentially die from it. Charles believed “it's better to get a vaccine rather than getting COVID,” even though he fully acknowledged the potential side effects that vaccines may trigger. Similarly, Elise was “concerned just about the long-term effects of this vaccine” and didn't “believe that it completely stopped COVID” but for him “avoiding hospitalization [due to COVID] was the biggest choice.” For vaccinated populations, “the risk of not getting the vaccine overweighted” the risk of unknown side effects, which lead to their vaccination decisions.

### Practical

Practical uncertainty refers to themes related to the processes and systems in place for administering the vaccines.

#### *System of care*

Most participants shared concerns about the potential cost of being unvaccinated, which could include concerns about unemployment, inaccessibility to certain services, or travel bans. Lorry and Janet expressed concerns about people getting fired because of their medical decisions. For Lorry, “the obstacles of not being able to travel… not going to concerts and the giveaways seem small to [her]”, and “the only big obstacle that [she sees] would be [her] job.” Janet shared her friends’ stories about being fired for making their own medical decisions. She made it clear that “making sure that [her] population is very safe is a top of [her] concern”, therefore “[she] was pretty much [sure] that she was going to get [vaccinated]”

Participants were concerned about more than just employment. Patrick shared a personal experience of being unable to access home care after having a toe amputated because he was not vaccinated.

Back on Labor Day of last year, I actually ended up in the hospital for unrelated - I had to have a toe amputated due to it being infected… after I got out of the hospital, I had a home care worker coming into my home and they basically said, because you're receiving home care, they're strong - they really wanted me [to get vaccinated]... otherwise I would lose my home care worker once every few days to help me with some things and change the bandages. So I knew that I had to have it because of, you know, the other problem I was dealing with. (Patrick, 53-year-old, vaccinated)

Janet mentioned that her friends’ children cannot play hockey because of their vaccination status which triggered Janet’s concerns about what will happen if her son is in the “must-vaccinated” age group. Interestingly, two participants shared their experiences using the potential cost of being unvaccinated to pose pressure on their vaccinated family members. Patrick made it clear to his nephew that he cannot go to Finland if he is not vaccinated, which nudged his nephew to overcome the safety concern and decide to get vaccinated. Esther’s daughter had “no other choice” but to get vaccinated to see her sister who went to school in London and got vaccinated.
