# Supplementary material for: Navigating the uncertainty: A novel taxonomy of vaccine hesitancy in the context of COVID-19
Source: PLoS One. 2023 Dec 21;18(12):e0295912. doi: 10.1371/journal.pone.0295912 (PMC10734916; doi:10.1371/journal.pone.0295912)
Supplement: S3 Appendix — (DOCX) [file pone.0295912.s003.docx]

**S3 Appendix: Focus Group Guide**

**Interview Guide for Focus Groups**

**GENERAL QUESTIONS**

- How has the COVID-19 pandemic affected you personally?

**TRUST QUESTIONS**

- Where have you been getting your main information about the pandemic and vaccine research and
- roll out?
- PROBE: If unresponsive, list of prompts:
- Social media
- Government websites, i.e. PHAC, Health Canada, Quebec public health
- News reports
- Friends and family
- Physician, nurse, pharmacist
- Overall, do you feel you have enough information to make confident decisions about your health
- and the vaccines?
- If you are not totally confident, what would it take to increase your confidence?

**VACCINE SAFETY, ACCESS, & HESITANCY**

- In general, how safe are vaccines?
- How safe do you think the COVID vaccines are?
- PROBE: What has influenced your ideas about the safety (or lack thereof) of COVID
- vaccines?
- PROBE: What would change your mind?
- Can you name a concrete venue (social media post, newspaper article, etc) that has influenced your ideas about the safety (or lack thereof COVID vaccines)?
- There have been some media reports that one-third Canadians are reluctant to take a COVID vaccine. How much do you share in these concerns?

**FOR UNVACCINATED GROUP:**

- How likely is it that you will get a COVID vaccine (once one becomes available to you)?
- Likely/very likely: What are the reasons why you want to be vaccinated? -
- Unlikely/very unlikely: What are the reasons why you do not want to be vaccinated?
- Unsure: What are the reasons why you are unsure about getting vaccinated?
- All : Have you experienced any limitation in access to vaccines that prevents you to get
- vaccinated?
- Do the following government initiatives change your opinion, making it more or less
- likely to get vaccinated?
- -Lotteries/Cash Prizes/Scholarships
- -Implementation of a COVID passport for non-essential activities
- -Travel restrictions for unvaccinated citizens

**FOR VACCINATED GROUP:**

- What are the reasons why you decided to get vaccinated
- What were the reasons why you were unsure about getting vaccinated?
- How easy or hard was it for you to get vaccinated? Did you experience any
- limitation in access to vaccines that delayed your vaccination?
- Did the following government initiatives change your opinion or influenced in your decision
- to get vaccinated?
- -Lotteries/Cash Prizes/Scholarships
- -Implementation of a COVID passport for non-essential activities
- -Travel restrictions for unvaccinated citizens

**SOCIAL ACCEPTABILITY**

- How did people around you take your decision to get/to not get vaccinated?
- Do you feel they accepted your decision? Has this generated problems in your social circle?

**CLOSING**

- Is there anything related to the pandemic or COVID vaccines that we haven’t talked about but that you think is important for us to know?
